# Global Habitat Suitability and Ecological Niche Separation in the Phylum Placozoa

**DOI:** 10.1371/journal.pone.0140162

**Published:** 2015-11-18

**Authors:** Omid Paknia, Bernd Schierwater

**Affiliations:** 1 ITZ, Ecology and Evolution, TiHo Hannover, Hannover, Germany; 2 Department of Ecology and Evolutionary Biology, Yale University, New Haven, Connecticut, United States of America; 3 Sackler Institute for Comparative Genomics and Division of Invertebrate Zoology, American Museum of Natural History, New York, New York, United States of America; Consiglio Nazionale delle Ricerche (CNR), ITALY

## Abstract

The enigmatic placozoans, which hold a key position in the metazoan Tree of Life, have attracted substantial attention in many areas of biological and biomedical research. While placozoans have become an emerging model system, their ecology and particularly biogeography remain widely unknown. In this study, we use modelling approaches to explore habitat preferences, and distribution pattern of the placozoans phylum. We provide hypotheses for discrete ecological niche separation between genetic placozoan lineages, which may also help to understand biogeography patterns in other small marine invertebrates. We, here, used maximum entropy modelling to predict placozoan distribution using 20 environmental grids of 9.2 km^2^ resolution. In addition, we used recently developed metrics of niche overlap to compare habitat suitability models of three genetic clades. The predicted distributions range from 55°N to 44°S and are restricted to regions of intermediate to warm sea surface temperatures. High concentrations of salinity and low nutrient concentrations appear as secondary factors. Tests of niche equivalency reveal the largest differences between placozoan clades I and III. Interestingly, the genetically well-separated clades I and V appear to be ecologically very similar. Our habitat suitability models predict a wider latitudinal distribution for placozoans, than currently described, especially in the northern hemisphere. With respect to biogeography modelling, placozoans show patterns somewhere between higher metazoan taxa and marine microorganisms, with the first group usually showing complex biogeographies and the second usually showing “no biogeography.”

## Introduction

**Fig 1:**
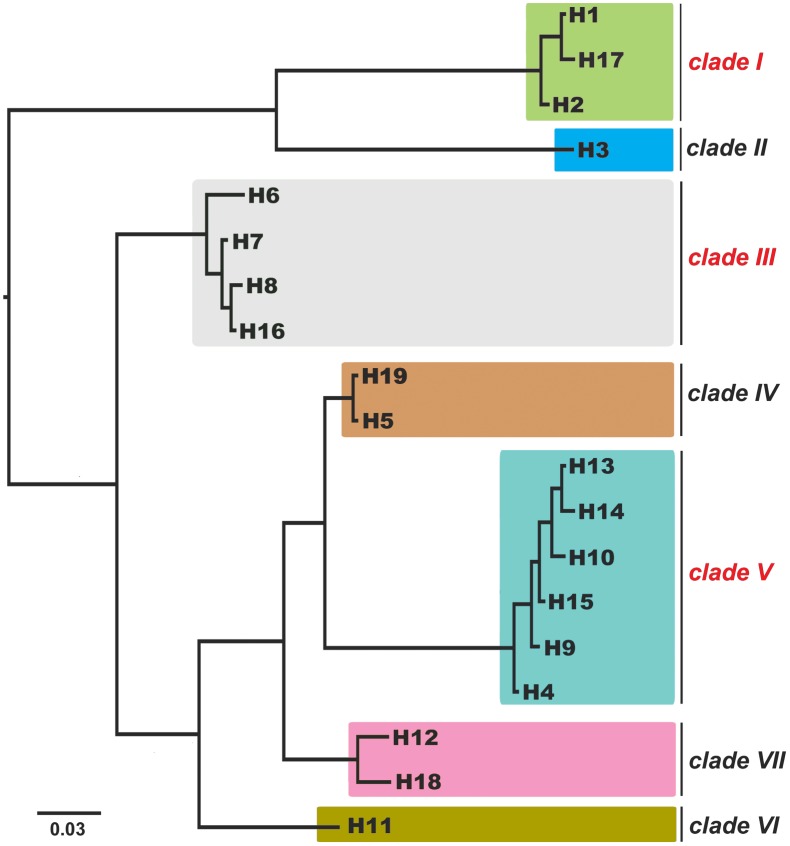
The phylogram of placozoan haplotypes (species) based on 16S sequences and Bayesian inference (Modified after Eitel et al [7]). The three clades highlighted in red have been investigated this study.

Placozoans are one of the most enigmatic groups of marine invertebrates, and they hold a key position in the metazoan Tree of Life [1]. Placozoans are small (1–3 mm) amoeba-like looking benthic animals with no organs, no symmetry and no specialized nerve or muscle cells ([2], but see [3]). Since its discovery in the late 19^th^ century [4] *Trichoplax adhaerens* has remained the only formally described placozoan species [5, 6]. Recent genetic studies, however, have revealed substantial genetic variation between more than a dozen clades, highlighting substantial diversification within this phylum. According to present knowledge the phylum consists of at least 19 species, which are here referred to 19 “haplotypes” forming at least seven well-separated clades ([7] See Fig 1). Species descriptions have proven to be extreme difficult due to the simple morphology of placozoans, which offers very few characters (but see [8]).

While a large body of knowledge has accumulated on the genetics of this phylum (e.g. [6], [9], [10]), very little is known about the ecology and biogeography of placozoans. Our understanding is extremely poor with respect to the interaction between placozoans and their environment. A small body of field studies provides some rough clues about the preferred habitats. Placozoans have been found in calm coastal waters only, suggesting that they avoid deep areas of strong currents (c.f. [11]). They have been found in shallow water but also in 20 m depth. Temperature and salinity have been hypothesized to be two most important limiting factors of their distribution [7]. Placozoans are known from all three oceans (i.e. the Atlantic, the Pacific, and the Indian oceans) and the recorded latitudinal distribution ranges from 48°N to 35°S [7]. Members of some clades (especially clade I and V) show wide distributions and appear to be cosmopolitans [12]. Other clades show a more restricted distribution (e.g. clade III). Overall, the number of cosmopolitans was found to be three times higher than the number of endemics, an observation that may be linked to the small size of placozoans [13, 14]. The “everything is everywhere hypothesis” for microscopic organisms suggests that placozoans may have no biogeographies due to their small size and high abundance, which fuel high frequencies of dispersal and low frequencies of allopatric speciation and endemism [15].

We here use ecological niche modeling methodology to address the question which environmental factors control the distribution of placozoans at the global scale and whether there are differences in distribution potential between different genetic lineages. Ecological niche modelling has widely been used to determine habitat suitability of marine organisms (e.g. [16], [17], [18]). We apply environmental variables previously recognized as potentially influencing placozoan distribution at a high global resolution of 5-arc min (9.2 km^2^) (developed by [19]). Maps of predicted habitat suitability are provided for the three main placozoan clades with the aim of (i) obtaining fundamental information on where placozoans are likely to occur and (ii) identifying environmental factors that control placozoan distribution. We furthermore examine whether different clades (Clade I, III, and V) occupy identical niches.

## Material and Methods

### Placozoan presence data

**Fig 2:**
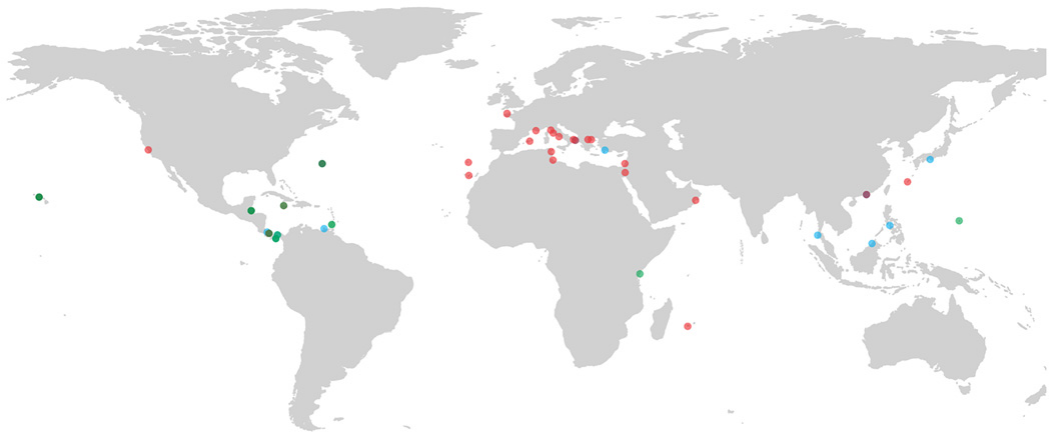
Global distribution of placozoans according to Eitel et al. [7] and unpublished data (see the text). Three red, green, and blue colors represent three investigated clades, clade I, III and V, respectively. Note that the number of localities on the map does not add to 79, because of points overlying in many localities.

The majority of placozoan haplotype field records used in this study are taken from Eitel *et al*. [7], who report a variety of locations and time periods across the globe, gathered from field work by the authors. In their paper, authors record placozoan haplotypes from numerous different localities. A small number of records has been taken from field work of our laboratory in 2012 and 2013, in Southern France (Niolon, Cassis, La Ciotat, Vieste, and Banyuls-sur-Mer). A total of 79 placozoans records was gathered and used for modelling habitat suitability (Fig 2). At the clade level, we restricted our analyses to three clades I, III, and V. We did not conduct analyses on other clades, as there are very low georeferenced records for those clades. For habitat suitability modelling at the clade level 28, 16 and 22, respectively, records of clades I, III and V could be used.

### Environmental data

An ever-increasing number of marine environmental data is becoming available, many of which are useful for habitat suitability modelling. We employed a global environmental dataset (Bio-ORACLE) for marine species distribution modelling recently provided as raster layers by Tyberghein *et al*. [19]. This data set has 5 arcmin (ca. 9 km^2^) resolution. In addition, a uniform landmask has been applied to all raster layers [19]. The uniform landmask procedure corrects discrepancies between coastline and environmental data by masking data pixels on land by calculating values for marine pixels without data by cubic extrapolation, improving data quality for fine scale coastal studies. This correction was essential for our study, as most records of placozoans are along coastlines. One disadvantage of the Bio-ORACLE dataset is the lack of multiple depth level variables. As we restricted our analyses to a narrow depth range (0–100 m), we anticipate that this shortcoming has no effect on our results. Since there is no concrete data on the interaction of placozoans with their habitats, at the beginning we considered and used a relatively large set of environmental variables, including 20 environmental covariates (Table 1). We reduced the number of the environmental predictors in the final model to avoid over-fitting models since modelling is generally negatively affected by incorporating too many predicting variables [20, 21]. Single-factor analysis is a popular approach for the selection of a smaller subset of available variables (e.g. [17]). We considered also the collinearity of variables to our pre-screening approach. Pearson’s correlation values higher than 0.75 is usually used as threshold of high collinearity [22, 23]. Thus, we modelled the habitat suitability based on variables that did not show high collinearity (pair-wise r_pearson_ < 0.75) but high relative contribution (> 0.75) to the models.

**Table 1:** List of environmental variables used in this study for modelling the global distribution of the phylum Placozoa. See Tyberghein *et al* [19] for full details of layers.

Variable	Description
**Remotely sensed data**	
Calcite concentration (mol/m3)	The concentration of CaCO_3_
Chlorophyll A concentration maximum (mg/m3)	The concentration of the photosynthetic pigment chlorophyll A
Chlorophyll A concentration mean (mg/m3)	
Chlorophyll A concentration minimum (mg/m3)	
Chlorophyll A concentration range (mg/m3)	
Diffuse attenuation coefficient at 490 nm (m -1) maximum	Indicator of water clarity
Diffuse attenuation coefficient at 490 nm (m -1) mean	
Diffuse attenuation coefficient at 490 nm (m -1) minimum	
Photosynthetically Available Radiation (Einstein/m2/day)Mean and maximum values	The quantum energy flux from the sun
Sea surface temperature (°C) maximum	The temperature of water at the ocean surface (topmost meter of the ocean water column).
Sea surface temperature (°C) mean	
Sea surface temperature (°C) minimum	
Sea surface temperature (°C) range	
**In situ measured oceanographic data**	
Dissolved oxygen (ml/l)	O2 concentration in water
Nitrate (μmole/l)	This layer contains both [NO_3_] and [NO_3_ + NO_2_] data
pH	Measure of the acidity
Phosphate (μmole/l)	Reactive ortho-phosphate concentration [HPO_4_^-2^]
Salinity (PSS)	Dissolved salt content
Silicate (μmole/l)	The concentration of silicate or ortho-silicic acid [Si(OH)4]

### Predicting habitat suitability

As all placozoan records have so far been collected from shallow waters close to seashores, we restricted the modelling of the habitat suitability of placozoans to shallow waters. A shallow-water mask was created from SRTM Plus bathymetry data [24] to restrict the analyses to areas of our interest, here shallow waters (≤ 100 m below the surface). All placozoan occurrence sites passed the mask. With this approach we limited the selection of background (pseudoabsences) samples to shallow water.

The MaxEnt modelling (maximum entropy modelling) approach was chosen to model habitat suitability for the phylum Placozoa and its three most speciose clades (Clade I, III, and V). MaxEnt is a presence-only approach, which estimates a probability distribution of maximum entropy, which is most spread out or closest to uniform. It is subject to restrictions imposed by the available (observed) occurrence records and environmental conditions across the study sites [25, 26]. This approach is suitable for the identification of new distributional areas in poorly known regions, even if the sample size is small [27]. In our study the main advantage of the presence-only data is that it releases us from the problem of unreliable absence records.

For model testing a subsample approach was applied in 100 model runs with 70% of data used in training and 30% retained as test points. Evaluation of the accuracy of predictive models is a critical step in ecological modelling. An increasing body of literature suggests that response curves to environmental variables are (at least for fundamental niches) unimodal [28]. Thus only linear and quadratic features were selected to build response curves. Despite recent criticism [29, 30], the Area Under the receiver-operator Curve (AUC) has stayed as the most popular approach for model evaluation in the MaxEnt literature, due to the lack of alternatives. AUC values range from 0 to 1, where 1 is a perfect fit. Useful models produce AUC values of 0.7–0.9, and excellent models produce AUC values above 0.9 [31]. The results can be reliable if key decisions about input data and settings of the MaxEnt are appropriately made [32]. By default MaxEnt assumes that all geographic spans have been equally sampled. However, our study shows a sampling bias towards the Mediterranean Sea. We accounted for this bias by providing a biased background layer covering of the Mediterranean region. With this layer we led MaxEnt to choose the background data with the same bias as the occurrence data. All three types of maps produced by MaxEnt, including raw, cumulative, and logistic, are related monotonically and ranked-based metrics for model fit (AUC) will be identical for them [33]. However, output types have different scales that lead to different visual maps and different interpretations. The literature recommends avoiding logistic output despite its popularity [32, 34]. We selected the raw type, as this form of output does not rely on post-processing assumptions [32].

### Niche equivalency

Several methods have been proposed for predicting ecological niche overlap (e.g. [35], [36]). The choice of the technique depends on the structure of the data and the hypothesis to be tested. We used *I*- and *D*-statistics to quantify the degree of similarity between habitat suitability models for the three clades using the “phyloclim” package of R. This analysis provides two tests: (i) niche equivalency (or identity) and (ii) background similarity. The niche equivalency test asks whether ecological niche models (here habitat suitability) of two species (here clades) are more different than expected if they were drawn from the same underlying distribution. The background similarity test asks whether habitat suitability models drawn from populations with partially or entirely non-overlapping distributions are any more different from one another than expected by random chance. Given the importance of geographical scale for investigating niche differentiation, the identity test corresponds to a small spatial scale (observed occurrence records of clades) relative to the background similarity test in which points are drawn from throughout a potential range. *D*-values range from 0 (niche models have no overlap) to 1 (niche models are identical). *I*-scores also range from 0 to 1 (no overlap to identical niche). For both tests 99 pseudoreplicate data sets were created from the pool occurrence data of each two clades in each niche comparison. For the equivalency test, habitat suitability models were estimated from a new set of environmental layer including all variables, which have been entered into multi-layer analyses. The *D*- and *I*-statistics were calculated on these niche models to produce a null distribution for comparison with the *D* and *I* scores estimated from the real data. For the background similarity test, comparisons were made by creating habitat suitability models based on random background cells chosen from the areas available for the two compared clades.

## Results

### Model evaluation

The MaxEnt models preformed well and discriminated suitable placozoans habitats according to the area under receiver operating characteristic curve (AUC) and threshold-based evaluation methods for four separate datasets (all-clades, clade I, clade III and clade V). All AUC values (for both training and test data) were higher than 0.90. A high level of uniformity among replications was indicated by low estimates of standard deviation among 100 model replicates for each data set (Table 2). The high AUC values were supported by high values of test gain and low omission rates, indicating that only few predicated presences were misclassified.

**Table 2:** Model evaluation statistics for MaxEnt models of four placozoans data sets (100 replications for each dataset): all-clades, Clade I, Clade III, Clade V.

Statistics	all-clades data	Clade I	Clade III	Clade V
**Evaluation**				
Average Training AUC	0.951	0.929	0.957	0.976
Average Test AUC	0.913	0.915	0.905	0.924
AUC _standard deviation_	0.024	0.048	0.033	0.026
Test gain	1.64	2.41	1.05	1.25
Prevalence	0.06	0.06	0.05	0.03
Entropy	7.01	7.08	6.52	5.92
**Threshold**				
Omission rate (threshold 10)^1^	1.7%	1.3%	4.1%	3.1%
Logistic threshold^2^	0.19	0.17	0.49	0.16
**Variable contribution (%)**				
Calcite mean	4.9	5.5	-	-
Chlorophyll A mean	3.2	-	-	4.8
Chlorophyll A range	-	10.4	-	-
Diffuse coefficient mean	-	-	-	20.4
Diffuse coefficient min	17.8	-	20	-
Dissolved oxygen	-	2.1	2.2	-
Nitrate mean	37.4	5.8	3.1	14.6
Phosphate Mean	11.4	17.3	27.4	16.3
Photosynthetically A. R. mean	-	5.9	-	-
Salinity mean	-	24.1	-	-
Silicate mean	-	0.7	5.6	-
Surface temp. max	7.2	8.1	-	43.9
Surface temp. mean	-	-	41.7	-
Surface temp. range	18.1	20.1	-	-

^1^ A threshold dependent omission rate (fixed value of 10).

^2^ The logistic threshold is based on equal test sensitivity and specificity test omission.

### Environmental variables

Temperature correlated negatively with dissolved oxygen (Pearson’s r: -0.97) and phosphate correlated positively with nitrate (Pearson’s r: 0.95). Maximum, mean, and minimum temperature are highly correlated. The same was found for Chlorophyll A concentration, and diffuse attenuation coefficient variables. Other variables show lower correlations (< 0.75). AUC values for each of 20 single-variable models for the all-clades dataset and each clade ranged from almost random (0.493) to highly distinguishable (0.887) (Table 3). The pH had the lowest score for the all-clades data set and clade I. The calcite showed the lowest score in clade III and the temperature range was the lowest in clade V. There was variation in AUC values between clades. Nevertheless, in each group there was one variable that outperformed the other variables. For the all-clades dataset, nitrate was the most explanatory variable. Mean salinity, mean surface temperature and mean diffuse coefficient were three most explanatory variables (Table 3).

**Table 3:** Test AUC values for MaxEnt models of the global distribution for three placozoan clades based on single variable analysis. Variable values in bold indicate those chosen for final multi-layer models after taking the collinearity values into account. Each column (data sets) uses five different sets of variables.

Variables	all-clades data	Clade I	Clade III	Clade V
Calcite mean	**0.750**	**0.797**	0.452	0.704
Chlorophyll A max	0.733	0.700	0.715	0.811
Chlorophyll A mean	**0.753**	0.696	0.667	**0.832**
Chlorophyll A min	0.752	0.724	0.598	0.821
Chlorophyll A range	0.619	**0.751**	0.678	0.668
Diffuse coefficient max	0.722	0.738	0.612	0.718
Diffuse coefficient mean	0.633	0.723	0.797	**0.852**
Diffuse coefficient min	**0.752**	0.734	**0.853**	0.851
Dissolved oxygen	0.665	**0.843**	**0.80**	0.712
Nitrate mean	**0.828**	**0.837**	**0.803**	**0.830**
pH mean	0.493	0.493	0.608	0.551
Phosphate Mean	**0.813**	**0.817**	**0.764**	**0.762**
Photosynthetically A. R. max	0.682	0.791	0.728	0.638
Photosynthetically A. R. mean	0.693	**0.799**	0.728	0.682
Salinity mean	0.689	**0.887**	0.454	0.500
Silicate mean	0.677	**0.765**	**0.765**	0.581
Surface temp. max	**0.768**	0.708	0.85	**0.835**
Surface temp. mean	0.693	**0.828**	**0.86**	0.736
Surface temp. min	0.611	0.733	0.812	0.726
Surface temp. range	**0.751**	**0.832**	0.581	0.458

### Multi-layer models

All explanatory variables, which provided AUC values higher than 0.75, were used to build a final multi-layer model. Although dissolved oxygen and nitrate variables showed high collinearity, we did not drop any of them in the final multi-layer model analyses because we assumed that, although highly correlated, these pair variables influence the biology of placozoans in different ways. Altogether, fourteen variables entered to four multi-layer models (see Table 2 for more details). All multi-layer models scored higher AUC values than any single-variable model. AUC values ranged from 0.905 (clade III) up to 0.924 (clade V) (Table 2). The models showed the dominance of nitrate, temperature and salinity in determining habitat suitability for all datasets. For the all-clades dataset nitrate, and surface temperature range contributed the most with minimum the diffuse attenuation coefficient coming next. For clade I, it was salinity that contributed the most to the model with surface temperature range in second place. For clades III and V, surface mean temperature and surface mean temperature were identified as the main variables (Table 2).

**Fig 3:**
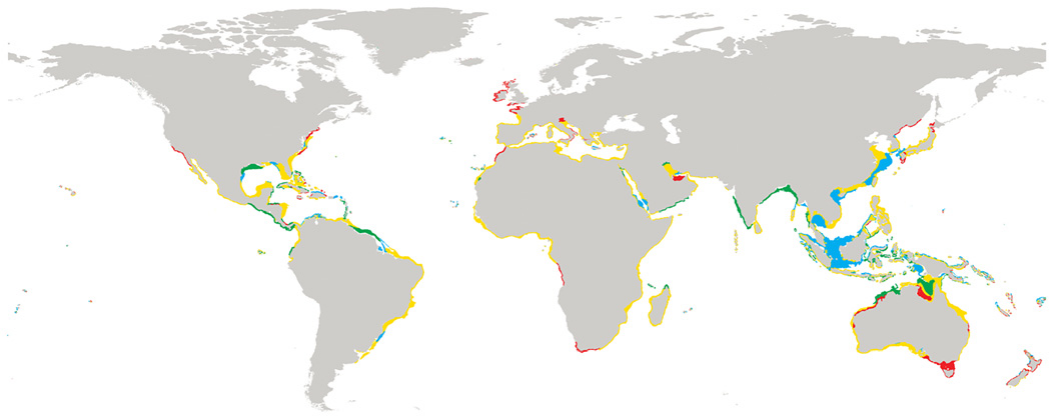
Global maps showing predicted habitat suitability for placozoans based on a 10^th^ percentile training presence threshold (see text). Three red, green, and blue colors represent the three placozoan clades, clade I, III and V, respectively. Records with black colors belong to other clades. Yellow represents regions where at least two clades overlap.

**Fig 4:**
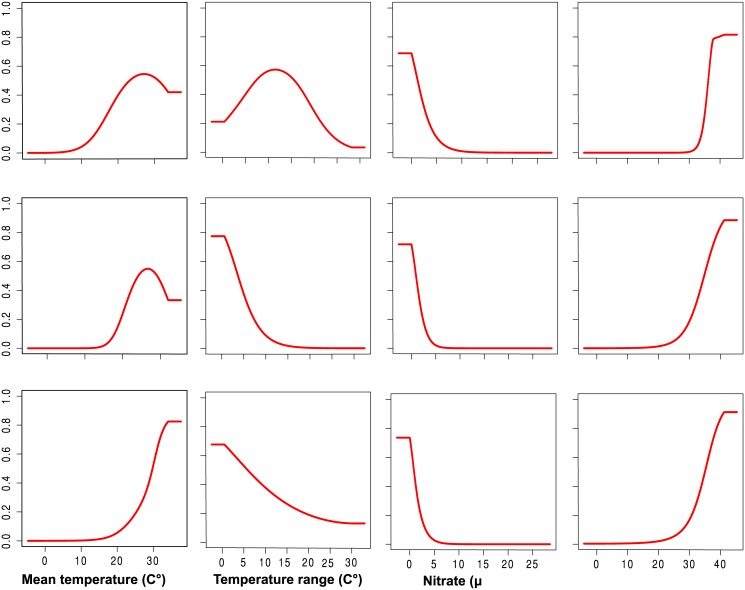
Occurrence probabilities for three placozoan clades in relation to four important abiotic factors, mean temperature, temperature range, nitrate, and salinity. Raw data of the occurrence probability can be found as supporting information (S1 Data).

The occurrence probability of placozoans was positively correlated with mean temperature and salinity. The highest probability of occurrence is in the warmest areas with the highest salinity concentration (Fig 3). In contrast, the occurrence probability decreases slowly with a decrease in temperature range. A sharp decrease of occurrence probability across nitrate dimension, suggests sensitivity of placozoans to nitrate concentration in ocean waters. The three clades showed different patterns of occurrence probability particularly across mean temperature and temperature range (see Fig 4).

### Habitat suitability

MaxEnt analyses suggest suitable habitats for placozoans throughout the world’s oceans, at a predicted distribution between 55°N and 44°S latitude. The majority of suitable habitats are predicted for the tropical Atlantic and the Central Indo Pacific (Fig 3). Other suitable habitats for the placozoans include the Indian Ocean, the temperate Northern Atlantic, the Mediterranean Sea, the Red Sea and the Persian Gulf (Fig 3). The model outputs for the three clades show differences between clades. Interestingly, all three clades show a preference for the Red Sea and the Persian Gulf. Clade I is largely predicted to be found in the Caribbean Sea, Northern Australia and temperate waters such as the Mediterranean Sea, the temperate Northern Pacific and the temperate Northern Atlantic (Fig 3). The majority of suitable habitats for clade III is restricted to the warm waters of the tropical Atlantic (37°N– 20°S), the tropical eastern Pacific and the central Indo-Pacific regions. Small parts of the southern Mediterranean, the Red Sea and the Persian Gulf are also predicted as suitable regions. In contrast, Clade V shows a very wide predicted latitudinal distribution. In addition to warm tropical waters, it is predicted to also appear in temperate waters such as the temperate coasts of Australia, South Africa, and South America.

### Niche equivalency

Observed *D* values of the niche equivalency tests range from 0.40 to 0.59, with clade I and III showing the largest differences while clade I and V were the most similar (Table 4). Two out of three pair-wise comparisons were significantly different from randomly chosen location points, while clades I and V are not being significantly different. The latter suggest that these two clades do not occupy different niches. Consistent with the low observed overlap between clade III and the two other clades, the hypothesis of niche equivalency between clade III and the other two clades was rejected because of a significant difference between observed and simulated niche overlap. Observed *I* statistics support the same trends seen in *D* statistics (*I* values ranging from 0.71 to 0.82). Niche overlap was the lowest between clades I and III and the highest between clades I and V. Again, differences between clades I and V were not significant. Background similarity results showed signals of niche differentiation between clades I and III in one direction. Two other comparisons suggest niche conservatism in both directions.

**Table 4:** Summary of niche equivalency and background similarity tests.

Comparison		
**Niche Identity test**		
Schoener’s Statistic	***D***	***P***
Clade I vs. Clade III	0.40	0.02
Clade I vs. Clade V	0.59	0.03
Clade III vs. Clade V	0.48	<0.01
Hellinger’s Statistic	***I***	***P***
Clade I vs. Clade III	0.71	<0.01
Clade I vs. Clade V	0.82	0.05
Clade III vs. Clade V	0.80	<0.01
**Background test**	**A vs. B**	**B vs. A**
Schoener’s Statistic	***D***	***D***
Clade I vs. Clade III	n.s.	Divergence
Clade I vs. Clade V	Conservatism	Conservatism
Clade III vs. Clade V	Conservatism	Conservatism
Hellinger’s Statistic	***I***	***I***
Clade I vs. Clade III	n.s.	Divergence
Clade I vs. Clade V	Conservatism	Conservatism
Clade III vs. Clade V	Conservatism	Conservatism

Significant values for niche equivalency indicate that two habitat suitability models are not identical/equivalent. Significant values for background similarity tests indicate that habitat suitability models are more similar than expected by chance. The background similarity tests contain two sets of results for each *I* and *D* statistics: a comparison of niche overlap between the observed occurrence of clade A and random points drawn from the background area of taxon B (A vs. B), or the converse (B vs. A). “Divergence” indicates that clades exhibit significant divergence (overlap is less than expected), while “Conservatism” indicates niche conservatism (overlap values are more similar than expected). NS indicates no significant difference between expected and observed overlap.

## Discussion

This study significantly improves our understanding of the global and regional distribution of placozoans and the factors determining habitat suitability for the phylum Placozoa. It appears that placozoan distribution is primarily restricted to regions of high sea surface temperature, with high salinity and low nutrient concentrations as secondary factors (see Fig 4). Levels of Chlorophyll A and pH appear as relevant minor factors (see Table 3). Temperature group variables were amongst the best performing for single-variable models (see Table 3) and agree widely with predictions of placozoans ecology [12]. Temperature group variables were also the major contributing factor to determine the final multivariable models for the tested clades (see Table 2). Temperature contributed decisively to final models of clade III and V, but not to the final model of clade I, suggesting that clades may show differential sensitivity for this important climate factor. For more than a century successful sampling of placozoans in the tropics and subtropics had fueled the hypothesis that this phylum occurs only in warm waters. The recent discovery of the placozoans in relatively cold waters of the English Channel has challenged this assumption ([37], also see [38]). Surprisingly, salinity was a relevant factor in the multi-model only for clade I. In the other three data sets salinity did not enter the final model of habitat suitability. This result suggests that salinity may be a less important environmental variable for the occurrence of placozoans than previously assumed. This finding is in agreement with recent field studies and laboratory observations that suggest a wide tolerance range of placozoans to salinity [7]. Nevertheless, it appears that a trade-off exists between salinity and temperature in their contribution. It is either one or other variable that plays a dominant role in distributional range model for each clade. Temperature group variables contributed largely to habitat suitability models of clade III and V while salinity was dominant factor for clade I. Our models suggest that phosphate and nitrate also play important roles for the occurrence and distribution of placozoans. This is not surprising given that concentration levels of inorganic nutrients, such as phosphate and nitrate, dictate population growth, diversity and species composition of microorganisms in the marine environment [39, 40]. Nitrate concentration is an important predictor of animal abundance through the development of algal turf consumed by herbivorous animals [41, 42]. Laboratory and field observations have shown that algae are the main energy resource for placozoans and placozoans are probably opportunistic grazers on algae and bacteria [5, 11].

Dissolved oxygen has been another variable that contributed in single-variable and the final multi-variable models. It has been shown that hypoxia (low oxygen condition) may act as an environmental stressor particularly for brackish water and seawater organisms, which are facing high costs of oxygen acquisition [43]. Surprisingly for the most simple metazoan animal, it has been shown that a functional hypoxic response system exists in placozoans [44]. These data suggest that placozoans have evolved under fluctuating oxygen level conditions and that this system has been developed to cope with hypoxia stress.

Habitat suitability models for all-clades predict a wider latitudinal distribution than currently known, especially in the northern hemisphere. The predicted distribution stretches toward the west coasts of England and Ireland (55°N). In the western Atlantic, we predict coastlines up to North Carolina and Virginia (40°N) as suitable habitats. In the eastern and western Pacific, predicted distribution stretches towards the San Francisco (USA) (39°N) and the Peter the Great Gulf (Russia) (42°N), respectively. The predicted distribution of placozoans in the southern hemisphere is a close match to the current distribution records (34°S). Tasmania Island coasts are the southernmost predicted suitable habitats in the southern hemisphere (44°N).

Habitat suitability maps produced in this study suggest placozoans presence in several regions that have not been sampled yet. Such regions are eastern and western Atlantic coasts, the west coast of the North America and Mesoamerica along the Pacific Ocean, the Persian Gulf and the Arabian Sea, India’s coastlines, east coast of Africa, and Madagascar. The northern and the southern Australia’s coastlines, and New Zealand remain as the major not survived regions in the Pacific.

One of the common challenges in distribution modelling studies, has been the transferability of models to new areas, in which sampling is sparse or non-existent [20, 45]. This is a critical issue especially when distribution modelling is used for predicting the effect of global climate change on a species’ distributional potential [20]. This might not be, however, the case for our study, where we created models from known occurrences of placozoans in temperate and tropical waters for discovering new populations/species in the same regions. Since we used the same environmental data to generate models and predictions, the background data are shared between training and prediction and the models do not have to be transferable [46].

Niche equivalency results presented in Table 4 highlight the similarity of niches for the two clades I and V. These two clades show no significant niche differentiation with respect to the selected environmental variables. Both clades appear to harbor widespread euryoecious species [7]. Both clades, however, differ significantly from clade III. Members of clade III have so far been found within a narrow latitudinal gradient only (26°N– 25°S), suggesting that their distribution is restricted to warm tropical and subtropical waters [7]. It must be noted here that the rejection of niche identity is necessary but not sufficient for the identification of environmental differentiation between two clades. Niche identity can be rejected also if lineages with identical niche requirements are distributed across a heterogeneous habitat [47]. Overall, our results suggest the existence of a structured biogeography for Placozoa. Placozoans somehow resemble a transition state between higher metazoan taxa and marine microorganisms, with the first group usually showing complex biogeographies and the second usually showing “no biogeography”.

Although our study provides a foundation for understanding the environmental variables that control placozoan distribution at a global scale, we acknowledge that the main environmental variables investigated in this study may covary with other unmeasured variables. That is, their effect may be indirect effect of unmeasured variables. For example, one gap in our predictor set might be the lack of information on the distribution range and density of microalgae (Microphytes), which could be main food sources for placozoans in the field. Recent studies emphasize important roles for temperature and nutrients as the main driving factor for the global richness of algae [48, 49].

The limited number of placozoan records for clades III, and V highlights the need for more targeted sampling. The habitat suitability results presented in this study are not meant to predict placozoan occurrences with pinpoint accuracy. These models must be useful however in directing research efforts to regions that have highest probability for placozoan presence. With increasing sampling, model prediction can be further improved and such data will clearly help to better understand the ecology of the enigmatic placozoans.

## Supporting Information

S1 Data Occurrence probability raw data. Raw data used for Fig 4 can be found in text files. (ZIP)
